# Supplementary material for: The ATF2/miR-3913-5p/CREB5 axis is involved in the cell proliferation and metastasis of colorectal cancer
Source: Commun Biol. 2023 Oct 10;6:1026. doi: 10.1038/s42003-023-05405-w (PMC10564889; doi:10.1038/s42003-023-05405-w)
Supplement: Supplementary file 2 — Supplementary Information [file 42003_2023_5405_MOESM2_ESM.pdf]

Supplementary information

The ATF2/miR-3913-5p/CREB5 axis is involved in the cell proliferation and metastasis of colorectal cancer

Weiyu Dai et al.

This file includes:

Supplementary figures

Supplementary tables

Supplementary Data Files

Supplementary figures

Supplementary Fig. 1

ONCOMIR

WashU Pan-Cancer miRNome Atlas

There are 7 cancer types where survival is significantly associated with hsa-miR-3913-5p

miRNA Name	Cancer Abbreviation	Log Rank P-value	Log Rank FDR	Z-score	Upregulated in:	Deceased Log2 Mean Expression	Living Log2 Mean Expression	T-Test P-value	T-Test FDR
hsa-miR-3913-5p	BLCA	1.83e-02	3.18e-01	2.309	Living	2.72	2.91	6.43e-01	8.41e-01
hsa-miR-3913-5p	HNSC	9.38e-03	5.64e-01	2.536	Living	1.34	1.60	1.99e-02	2.05e-01
hsa-miR-3913-5p	KICH	2.01e-02	3.08e-01	2.375	Deceased	1.65	0.92	8.59e-02	5.73e-01
hsa-miR-3913-5p	KIRC	4.80e-02	8.08e-01	2.012	Deceased	0.80	0.73	2.97e-01	4.56e-01
hsa-miR-3913-5p	LGG	5.56e-07	3.02e-03	5.488	Deceased	1.40	0.80	8.94e-02	3.02e-01
hsa-miR-3913-5p	READ	6.99e-04	5.18e-01	2.345	Living	0.08	0.37	1.48e-04	1.06e-01
hsa-miR-3913-5p	THYM	7.98e-04	1.47e-01	3.451	Living	2.10	3.00	1.99e-05	1.72e-04

BLCA: bladder urothelial carcinoma; **HNSC**: head and neck squamous cell carcinoma; **KICH**: kidney chromophobe; **KIRC**: kidney renal clear cell carcinoma; **LGG**: brain lower grade glioma; **READ**: rectal adenocarcinoma; **THYM**: thymoma

Supplementary Fig. 1 There are cancer types of which survivals were associated with miR-3913-5p.

Supplementary Fig. 2

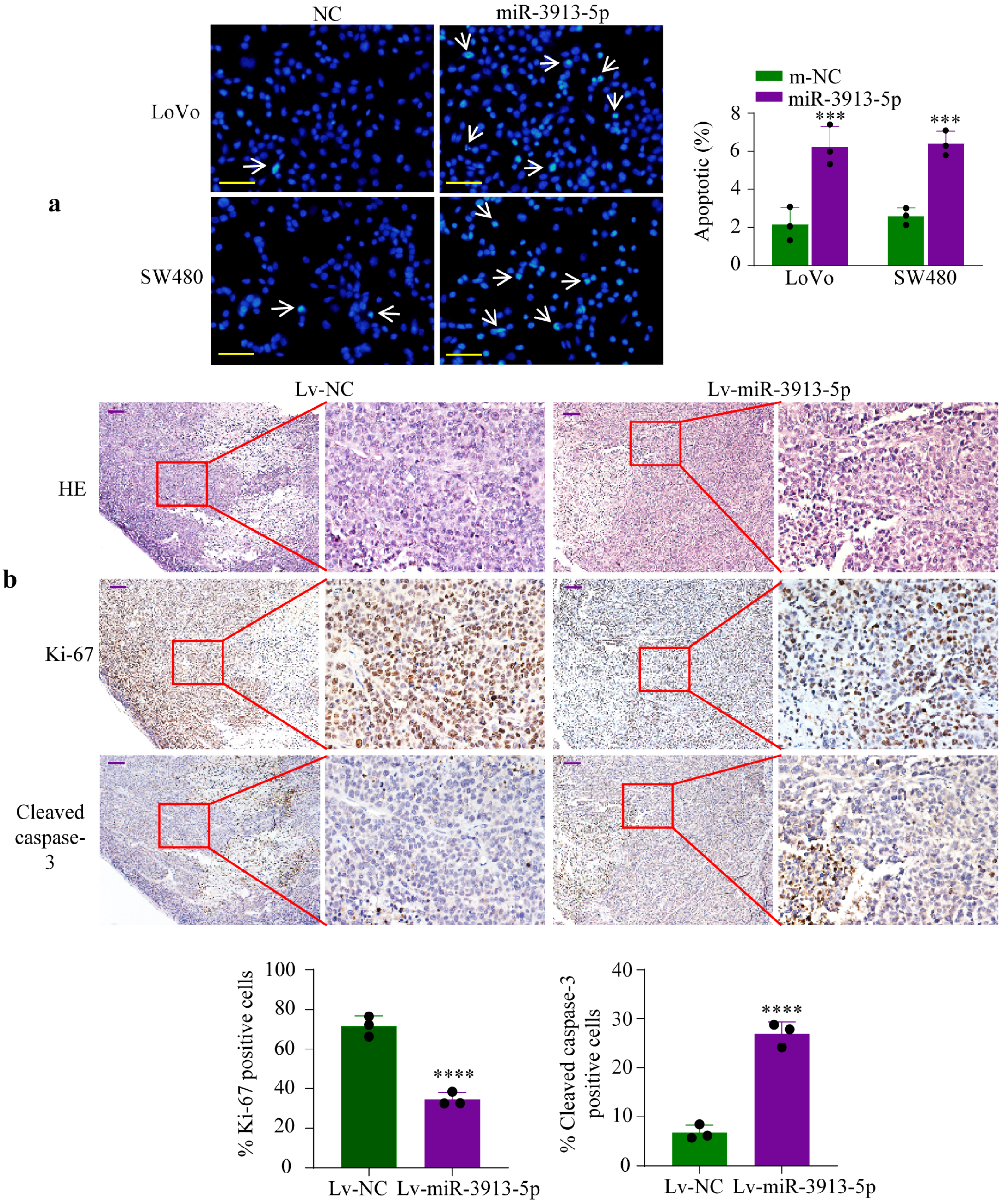

Supplementary Fig. 2 miR-3913-5p suppresses proliferation and induces cell apoptosis in CRC. a Apoptosis observed by Hoechst 33258 staining in CRC cells. Student's t-test; ***p<0.01. **b** IHC assays showed the Ki-67 and cleaved caspase-3 staining in subcutaneous tumors of nude mice. Student's t-test; ****p<0.001. Scale bars, 50 μm in (a), 100 μm in (b).

Supplementary Fig. 3

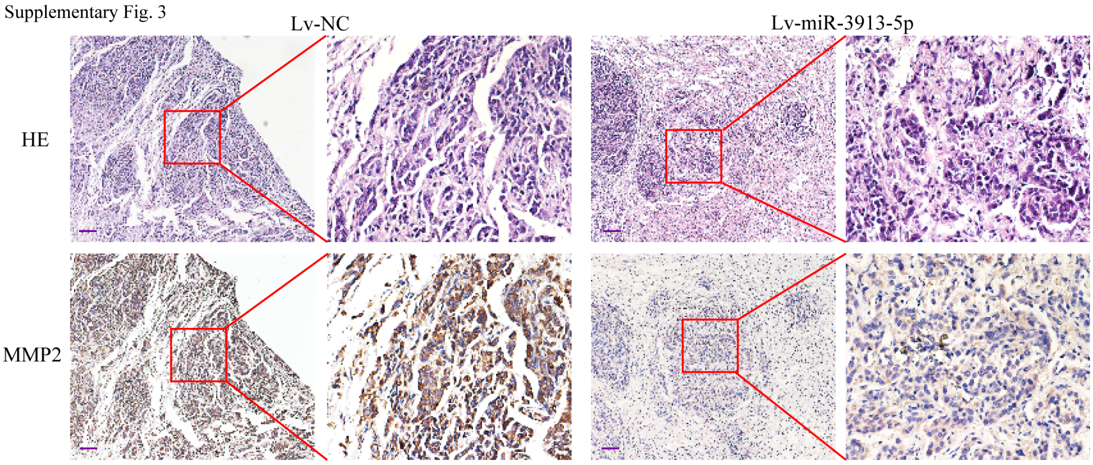

Supplementary Fig. 3 miR-3913-5p inhibits metastasis of CRC in vivo. MMP2 staining of lungs of nude mice was observed by IHC assays. Scale bars, 100 μm.

Supplementary Fig. 4

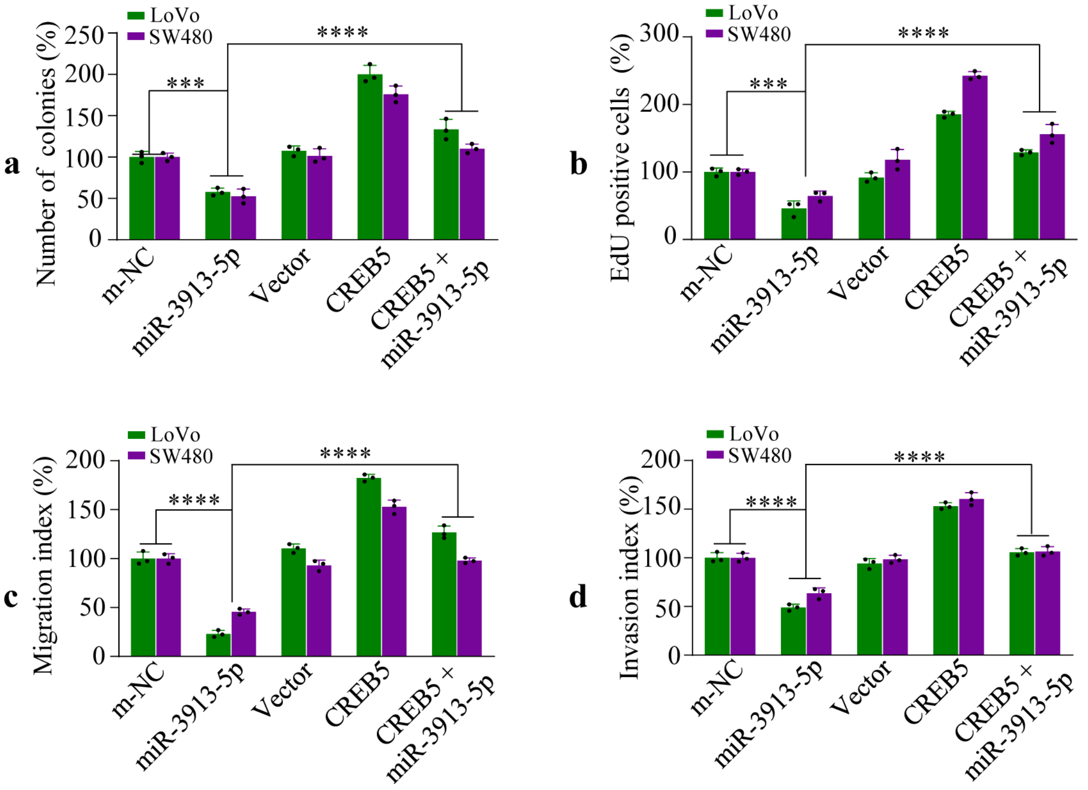

Supplementary Fig. 4 miR-3913-5p mediates CRC cell proliferation, migration and invasion by targeting CREB5. a-b Colony formation assays (a) and EdU assays (b) suggested that the inhibition of CRC cells proliferation induced by miR-3913-5p was inversed after overexpression of CREB5. Student's t-test; ***p<0.01; ****p<0.001. **c-d** Transwell assays indicated that suppression of CRC cells migration (c) and invasion (d) abilities by miR-3913-5p was counteracted after CREB5 administration. Student's t-test; ****p<0.001.

Supplementary Fig. 5

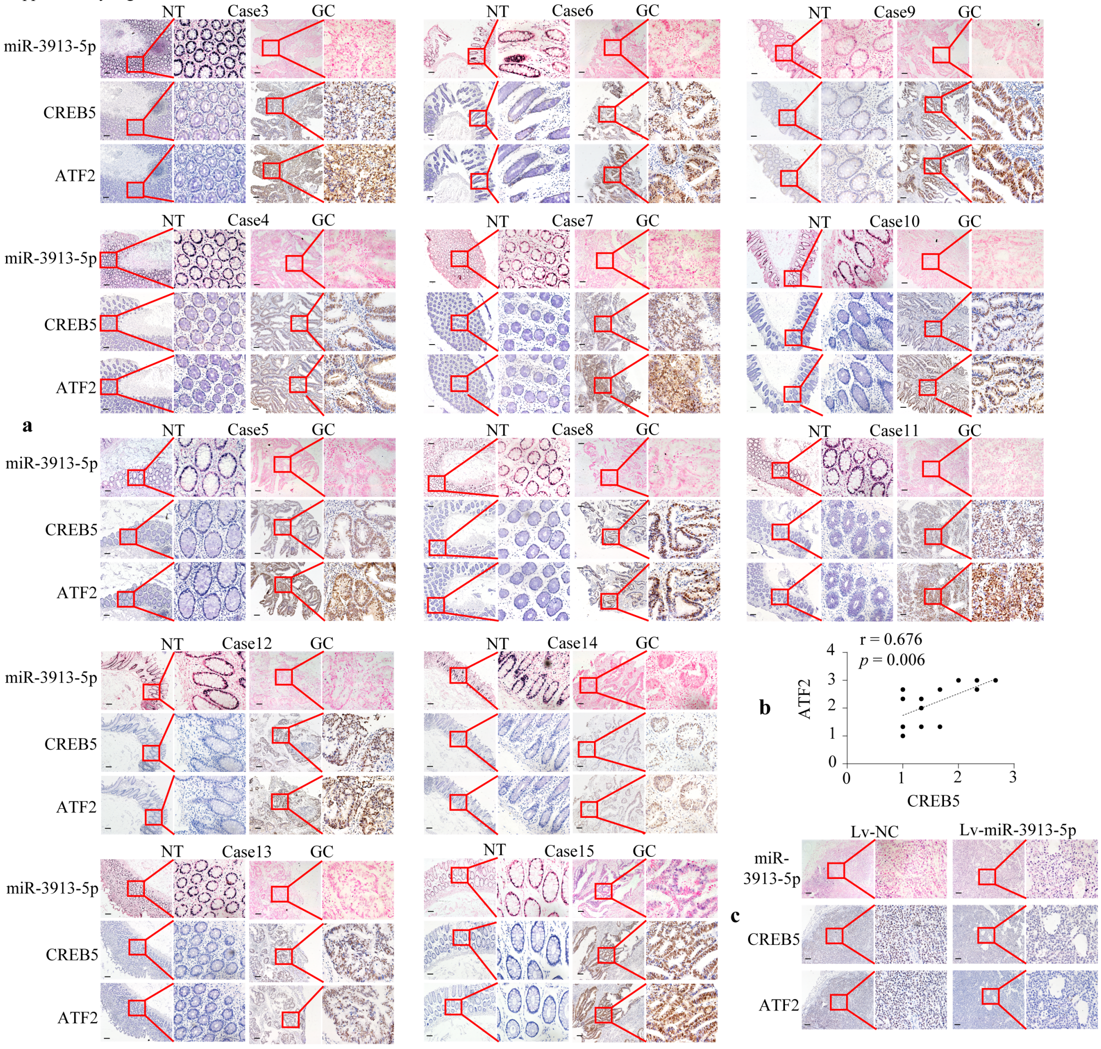

Supplementary Fig. 5 The miR-3913-5p, CREB5, ATF2 staining in CRC tissues and subcutaneous tumors of nude mice. a The miR-3913-5p, CREB5, ATF2 staining in CRC tissues were showed by ISH or IHC assays. **b** The correlation of CREB5 and ATF2 staining score in CRC tissues. **c** ISH and IHC assays displayed the miR-3913-5p, CREB5, ATF2 staining in subcutaneous tumors. Scale bars, 100 μm in (a, c) .

Supplementary Fig. 6

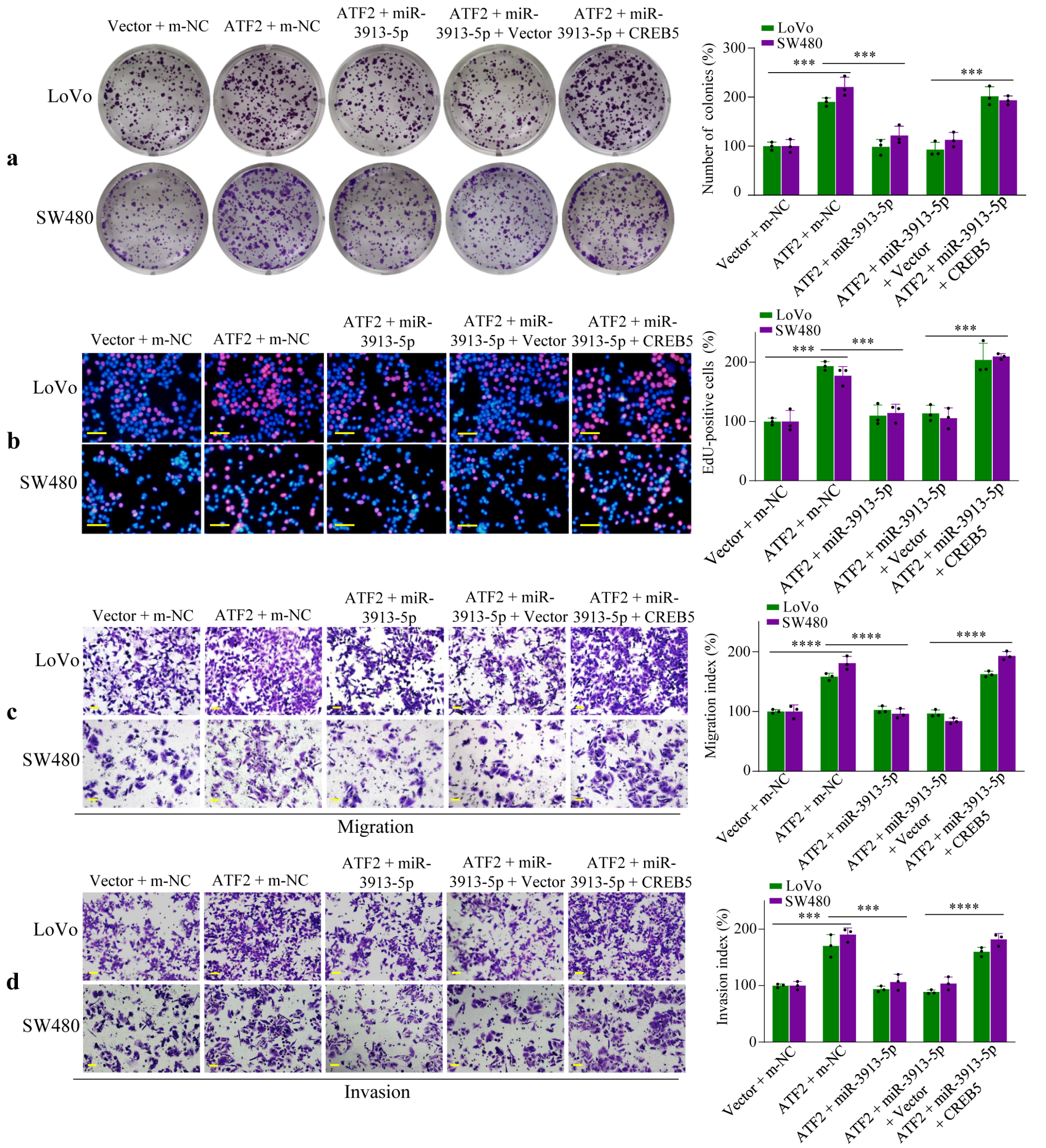

Supplementary Fig. 6 The function of ATF2/miR-3913-5p/CREB5 axis in CRC cells. a-d The colony formation (a), EdU (b) and transwell assays (c-d) showed that miR-3913-5p overexpression eroded the enhanced proliferation, migration and invasion by ATF2, and CREB5 overexpression could reversed these effects. Student's t-test; ***p<0.01; ****p<0.001. Scale bars, 50 μm in (b-d).

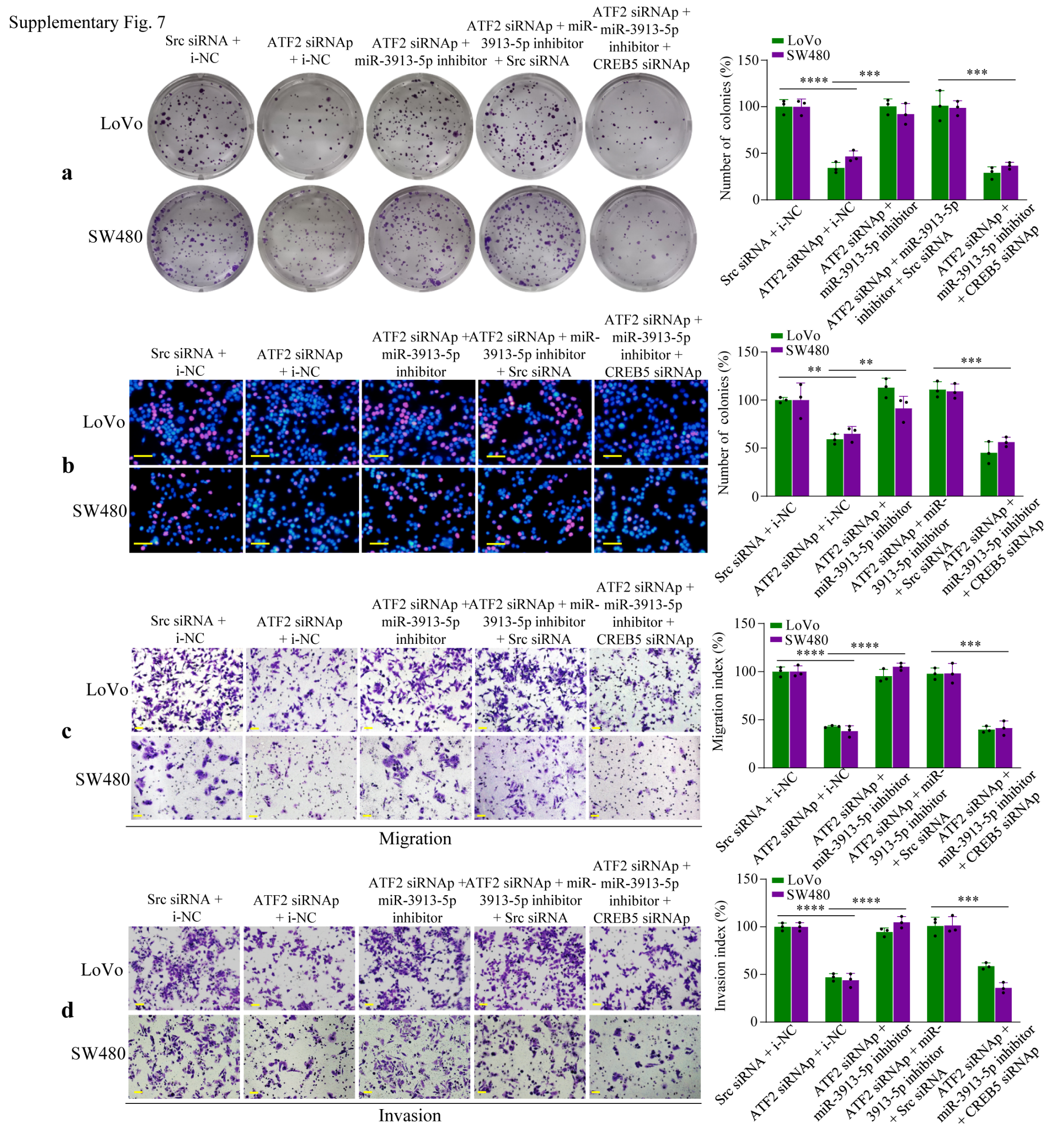

Supplementary Fig. 7 The function of CREB5 and ATF2 interaction in regulating miR-3913-5p. a-d The colony formation (a), EdU (b) and transwell assays (c-d) suggested that miR-3913-5p inhibition degraded the weakened proliferation, migration and invasion by ATF2 suppression, while CREB5 inhibition deteriorated the effects above. Student's t-test; ***p<0.01; ****p<0.001. Scale bars, 50 μm in (b-d).

Supplementary Fig. 8

Uncropped blots for Fig. 4c, d

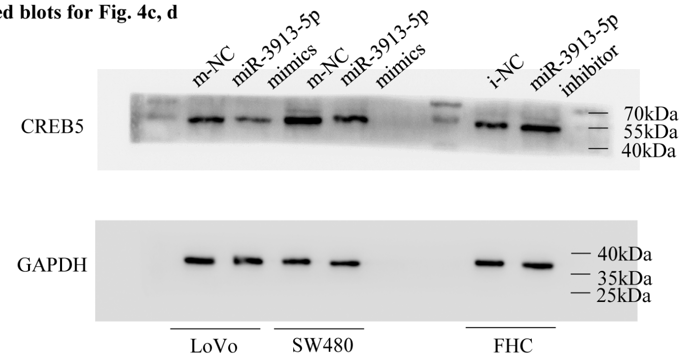

Uncropped blots for Fig. 5c

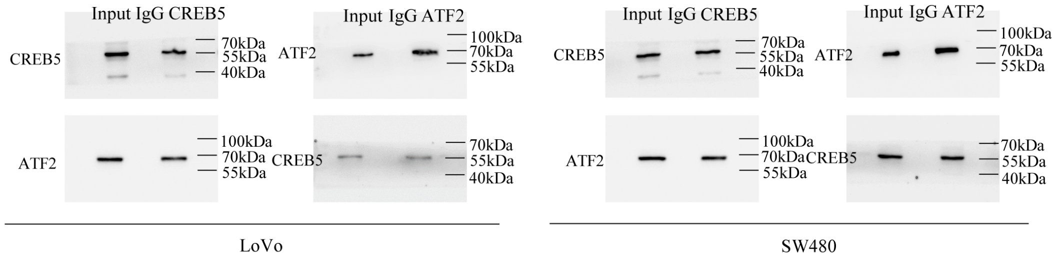

Uncropped blots for Fig. 7a

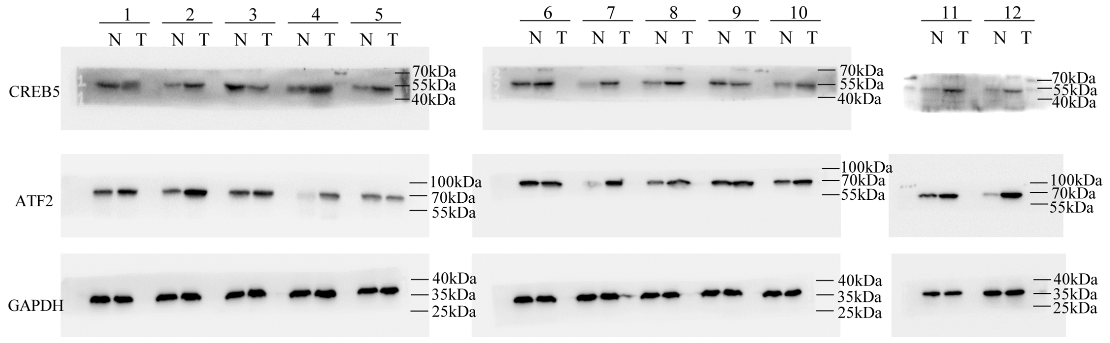

Supplementary Fig. 8 Uncropped blots for Fig. 4c, d, Fig. 5c, Fig .7a.

Supplementary Fig. 9

Full unedited gels for Fig. 5f

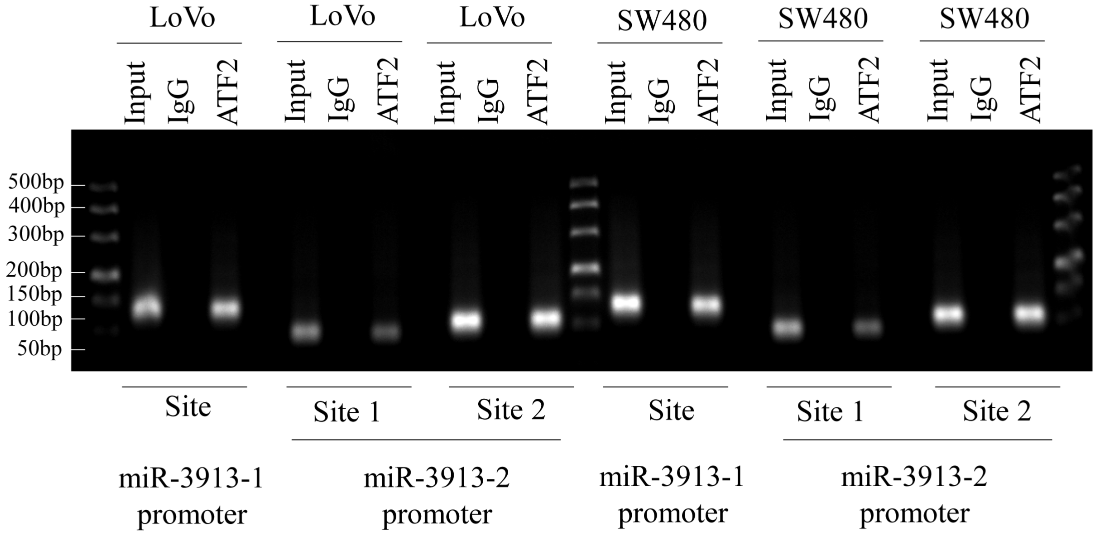

Supplementary Fig. 9 Full unedited gels for Fig. 5f.

Supplementary tables

Supplementary Table 1 miR-3913-5p expression levels of CRC patients in the different clinicopathological subgroups.

Parameters	Number	miR-3913-5p expression	*p*-value
**Gender**			0.632
Male	48	0.977 ± 0.799	
Female	43	0.897 ± 0.788	
**Age (yr)**			0.962
＜60	43	0.944 ± 0.678	
⩾60	48	0.936 ± 0.886	
**Tumor size (cm)**			0.485
＜3	15	1.070 ± 0.739	
⩾3	76	0.914 ± 0.802	
**Differentiation**			**0.035**
Well/Moderate	72	1.029 ± 0.838	
Poor	19	0.601 ± 0.444	
**Tumor invasion**			**0.002**
T1-T2	15	1.518 ± 1.077	
T3-T4	76	0.825 ± 0.673	
**Lymph node metastasis**			**＜0.001**
Absent	36	1.698 ± 0.743	
Present	55	0.443 ± 0.217	
**Distant metastasis**			**0.001**
Absent	82	0.144 ± 0.061	
Present	9	5.578 ± 4.363	
**TNM stage**			**＜0.001**
I - II	35	1.741 ± 0.707	
III - IV	56	0.438 ± 0.217	

Supplementary Table 2 miR-3913-5p expression levels of GC patients in the different clinicopathological subgroups.

Parameters	Number	miR-3913-5p expression	*p*-value
**Gender**			0.514
Male	60	1.356 ± 2.907	
Female	20	0.913 ± 1.392	
**Age (yr)**			0.213
＜60	37	1.639 ± 3.623	
⩾60	43	0.907 ± 1.148	
**Tumor size (cm)**			0.767
＜5	42	1.162 ± 1.646	
⩾5	38	1.337 ± 3.396	
**Differentiation**			**0.021**
Well/Moderate	21	2.370 ± 4.613	
Poor	59	0.845 ± 1.151	
**Tumor invasion**			**＜0.001**
T1-T2	14	3.682 ± 5.295	
T3-T4	66	0.729 ± 1.062	
**Lymph node metastasis**			**0.001**
Absent	16	3.103 ± 5.125	
Present	64	0.781 ± 1.095	
**Distant metastasis**			0.296
Absent	73	1.340 ± 2.714	
Present	7	0.254 ± 0.340	
**TNM stage**			**0.004**
I - II	25	2.471 ± 4.148	
III - IV	55	0.688 ± 1.145	

Supplementary Table 3 CREB5 expression levels of CRC patients in the different clinicopathological subgroups.

Parameters	Number	CREB5 expression	*p*-value
**Gender**			0.713
Male	48	4.055 ± 4.507	
Female	43	3.662 ± 5.637	
**Age (yr)**			0.162
＜60	43	3.085 ± 3.649	
⩾60	48	4.572 ± 5.985	
**Tumor size (cm)**			0.225
＜3	15	2.418 ± 2.346	
⩾3	76	4.156 ± 5.389	
**Differentiation**			0.056
Well/Moderate	72	3.351 ± 3.843	
Poor	19	5.832 ± 7.999	
**Tumor invasion**			0.182
T1-T2	15	2.274 ± 3.409	
T3-T4	76	4.184 ± 5.272	
**Lymph node metastasis**			**<0.001**
Absent	36	1.467 ± 1.433	
Present	55	5.441 ± 5.896	
**Distant metastasis**			**<0.001**
Absent	82	3.228 ± 3.828	
Present	9	9.713 ± 9.776	
**TNM stage**			**<0.001**
I - II	35	1.407 ± 1.407	
III - IV	56	5.408 ± 5.848	

Supplementary Table 4 ATF2 expression levels of CRC patients in the different clinicopathological subgroups.

Parameters	Number	ATF2 expression	*p*-value
**Gender**			0.643
Male	48	1.756 ± 1.203	
Female	43	1.649 ± 0.976	
**Age (yr)**			0.382
＜60	43	1.599 ± 1.125	
⩾60	48	1.801 ± 1.074	
**Tumor size (cm)**			0.481
＜3	15	1.522 ± 0.908	
⩾3	76	1.742 ± 1.133	
**Differentiation**			0.070
Well/Moderate	72	1.599 ± 0.896	
Poor	19	2.110 ± 1.622	
**Tumor invasion**			0.082
T1-T2	15	1.255 ± 0.992	
T3-T4	76	1.794 ± 1.101	
**Lymph node metastasis**			**0.011**
Absent	36	1.345 ± 0.683	
Present	55	1.941 ± 1.249	
**Distant metastasis**			**0.016**
Absent	82	1.614 ± 1.064	
Present	9	2.537 ± 1.099	
**TNM stage**			**0.008**
I - II	35	1.323 ± 0.679	
III - IV	56	1.945 ± 1.238	

Supplementary Table 5 The primers used in this study.

Experiment	Name	Position or orientation	Sequence (5’-3’)
qPCR	CREB5	F	CCCTGCCCAACCCTACAATG
		R	GGACCTTGCATCCCCATGAT
	ATF2	F	AATTGAGGAGCCTTCTGTTGTAG
		R	CATCACTGGTAGTAGACTCTGGG
	TRPS1	F	AGCCCCAGTAAGGGAGGAAA
		R	GGGTGCAGGCCATATCTTGAG
	SMAD2	F	CCGACACACCGAGATCCTAAC
		R	GAGGTGGCGTTTCTGGAATATAA
	CDH12	F	TTTGATGGAGGTCTCCTAACACC
		R	ACGTTTAACACGTTGGAAATGTG
	NRF1	F	AGGAACACGGAGTGACCCAA
		R	TATGCTCGGTGTAAGTAGCCA
	ATF7	F	GTTCCCCACCACTGAAGGAG
		R	ATGGTGGAGCCTGTGTGATG
	BATF3	F	GCCAAACTCACAGAGCCCTT
		R	TACTAGCTGCCAGGGTGGAT
	GAPDH	F	AAATCCCATCACCATCTTCC
		R	TCACACCCATGACGAACA
	mature miR-3913-5p	Cat. No: HmiRQP1990, purchased from GeneCopoeia (Guangzhou, China)	
	snRNA U6	Cat. No: HmiRQP9001, purchased from GeneCopoeia (Guangzhou, China)	
Chip (MIR3913-1 promoter	Site	F: -1708~ -1688	CACGCCCGGCTAATTTTTGT
		R: -1616~ -1596	TACTTTGGGAGGCCGAGGTA
Chip (MIR3913-2 promoter)	Site 1	F: -224~ -204	GTTTCACCATGTTGGCCAGG
		R: -150~ -130	GCTCACCCCTGTAATCCCAG
	Site 2	F: -1957 ~ -1937	ATATAGGTGTGGTGGCTCAT
		R: -1898 ~ -1877	GAACTTCTGACCTCAAGCAAT

Supplementary Table 6 The detailed sequences of the miRNA mimics, miRNA inhibitor and siRNAs.

**Name**	**Orientation**	**Sequence (5'-3')**
m-NC	sense	5'-UUCUCCGAACGUGUCACGUTT-3'
	antisense	5'-ACGUGACACGUUCGGAGAATT-3'
miR-3913-5p mimics	sense	5'-UUUGGGACUGAUCUUGAUGUCU-3'
	antisense	5'-ACAUCAAGAUCAGUCCCAAAUU -3'
i-NC	Sense	5'-CAGUACUUUUGUGUAGUACAA-3'
miR-3913-5p inhibitor	Sense	5'-AGACAUCAAGAUCAGUCCCAAA-3'
Scr siRNA (Negative control)	sense	5'-UUCUCCGAACGUGUCACGUTT-3'
	antisense	5'-ACGUGACACGUUCGGAGAATT-3'
ATF2 siRNA1	sense	5'-CCUCUUGCAACACCUAUCATT -3'
	antisense	5'-UGAUAGGUGUUGCAAGAGGTT -3'
ATF2 siRNA2	sense	5'-CCUGUGGAAUAUGAGUGAUTT-3'
	antisense	5'-AUCACUCAUAUUCCACAGGTT -3'
ATF2 siRNA3	sense	5'-GGAAGUACCAUUGGCACAATT-3'
	antisense	5'-UUGUGCCAAUGGUACUUCCTT -3'
ATF7 siRNA1	sense	5'-CCAUGGUGCCCAACAUUCCUGGUAU-3'
	antisense	5'-AUACCAGGAAUGUUGGGCACCAUGG-3'
ATF7 siRNA2	sense	5'-CUGUGAGGAAGUGGGGCUCTT-3'
	antisense	5'-GAGCCCCACUUCCUCACAGTT-3'
ATF7 siRNA3	sense	5'-GGCAGGUUAAAGGAGCUUATT-3'
	antisense	5'-UAAGCUCCUUUAACCUGCCTT -3'
BATF3 siRNA1	sense	5'-GCUCAGAGAAGUCGGAAGATT-3'
	antisense	5'-UCUUCCGACUUCUCUGAGCTT-3'
BATF3 siRNA2	sense	5'-CUAUGAACUUUGUGCCAGUTT-3'
	antisense	5'-ACUGGCACAAAGUUCAUAGTT-3'
BATF3 siRNA3	sense	5'-CCUCUAGAAUUUGGAUAAUTT-3'
	antisense	5'-AUUAUCCAAAUUCUAGAGGTT-3'
CREB5 siRNA1	sense	5'-GCGGAAUAUCUCGAUGCAUTT-3'
	antisense	5'-AUGCAUCGAGAUAUUCCGCTT-3'
CREB5 siRNA2	sense	5'-CCUACAAUGCCAGGAUCUUTT-3'
	antisense	5'-AAGAUCCUGGCAUUGUAGGTT-3'
CREB5 siRNA3	sense	5'-CCAUGUCAAAUGGGAACAUTT-3'
	antisense	5'-AUGUUCCCAUUUGACAUGGTT-3'

Supplementary Data Files

Supplementary Data File 1 All source data underlying the graphs and charts showed in the figures.
