# Supplementary material for: The ATF2/miR-3913-5p/CREB5 axis is involved in the cell proliferation and metastasis of colorectal cancer
Source: Commun Biol. 2023 Oct 10;6:1026. doi: 10.1038/s42003-023-05405-w (PMC10564889; doi:10.1038/s42003-023-05405-w)
Supplement: Supplementary file 3 — Description of Additional Supplementary Data [file 42003_2023_5405_MOESM3_ESM.docx]

**Description of Additional Supplementary Files**

**File name:** Supplementary Data 1

**Description:** All source data underlying the graphs and charts showed in the figures.
