# Supplementary material for: The ATF2/miR-3913-5p/CREB5 axis is involved in the cell proliferation and metastasis of colorectal cancer
Source: Commun Biol. 2023 Oct 10;6:1026. doi: 10.1038/s42003-023-05405-w (PMC10564889; doi:10.1038/s42003-023-05405-w)
Supplement: Supplementary file 5 — Reporting summary [file 42003_2023_5405_MOESM5_ESM.pdf]

nature portfolio

Corresponding author(s): Jide Wang, Miaomiao Pei & Jiaying Li

Last updated by author(s): Sep 29, 2023

Reporting Summary

Nature Portfolio wishes to improve the reproducibility of the work that we publish. This form provides structure for consistency and transparency in reporting. For further information on Nature Portfolio policies, see our Editorial Policies and the Editorial Policy Checklist.

Statistics

For all statistical analyses, confirm that the following items are present in the figure legend, table legend, main text, or Methods section.

n/a	Confirmed	
☐	☒	The exact sample size (*n*) for each experimental group/condition, given as a discrete number and unit of measurement
☐	☒	A statement on whether measurements were taken from distinct samples or whether the same sample was measured repeatedly
☐	☒	The statistical test(s) used AND whether they are one- or two-sided<br>*Only common tests should be described solely by name; describe more complex techniques in the Methods section.*
☐	☒	A description of all covariates tested
☐	☒	A description of any assumptions or corrections, such as tests of normality and adjustment for multiple comparisons
☐	☒	A full description of the statistical parameters including central tendency (e.g. means) or other basic estimates (e.g. regression coefficient) AND variation (e.g. standard deviation) or associated estimates of uncertainty (e.g. confidence intervals)
☐	☒	For null hypothesis testing, the test statistic (e.g. *F*, *t*, *r*) with confidence intervals, effect sizes, degrees of freedom and *P* value noted<br>*Give P values as exact values whenever suitable.*
☒	☐	For Bayesian analysis, information on the choice of priors and Markov chain Monte Carlo settings
☒	☐	For hierarchical and complex designs, identification of the appropriate level for tests and full reporting of outcomes
☐	☒	Estimates of effect sizes (e.g. Cohen's *d*, Pearson's *r*), indicating how they were calculated

Our web collection on statistics for biologists contains articles on many of the points above.

Software and code

Policy information about availability of computer code

Data collection	Data of EdU assay, Hoechst 33258 staining assay, Transwell assay, wound-healing assay, in situ hybridization and immunohistochemistry were collected using Olympus IX73 inverted microscope and Olympus BX53F upright research microscope. Quantitative real-time PCR data were collected using Roche LightCycler 480 II. The protein bands were visualized by using Tanon 5200CE Imaging Analysis System. Quantified relative promote activity was measured in a GloMax 20/20 Luminometer (Promega, Madison, WI, USA).
Data analysis	Statistical analyses were performed using IBM SPSS Statistics 23 and GraphPad Prism 8. Data of colony formation assays, EdU assay, Transwell assay, wound-healing assay and Hoechst 33258 staining assay were analyzed using Image J software.

For manuscripts utilizing custom algorithms or software that are central to the research but not yet described in published literature, software must be made available to editors and reviewers. We strongly encourage code deposition in a community repository (e.g. GitHub). See the Nature Portfolio guidelines for submitting code & software for further information.

Data

Policy information about availability of data

All manuscripts must include a data availability statement. This statement should provide the following information, where applicable:

- Accession codes, unique identifiers, or web links for publicly available datasets
- A description of any restrictions on data availability
- For clinical datasets or third party data, please ensure that the statement adheres to our policy

All data generated or analyzed during this study are included in the article and supplemental files, or available from the corresponding author on reasonable request. All source data underlying the graphs and charts showed in the figures are presented in Supplementary Data File 1. Uncropped and unedited blots/gels are presented in Supplementary Fig. 8-9.

Research involving human participants, their data, or biological material

Policy information about studies with human participants or human data. See also policy information about sex, gender (identity/presentation), and sexual orientation and race, ethnicity and racism.

Reporting on sex and gender	Male and female patients were randomly enrolled.
Reporting on race, ethnicity, or other socially relevant groupings	Human colorectal cancer samples, gastric cancer specimens and the paired noncancerous tissues were collected from Nanfang Hospital, Southern Medical University (Guangzhou, China). All patients whose tissue samples were collected in our study were Asians.
Population characteristics	Patients who were diagnosed with colorectal cancer or gastric cancer and underwent standard surgical treatment.
Recruitment	All patients were underwent standard surgical treatment and histopathological examination in Nanfang hospital, Southern Medical University (Guangzhou, China). Each sample was attached to a confirmed pathological diagnosis and was staged according to the 8th edition of the American Joint Committee on Cancer (AJCC) cancer staging manual. None of the patients has received any preoperative chemo/radiotherapy.
Ethics oversight	Experiments were conducted with the approval of the Medical Ethics Committee of Nanfang Hospital, Southern Medical University.

Note that full information on the approval of the study protocol must also be provided in the manuscript.

Field-specific reporting

Please select the one below that is the best fit for your research. If you are not sure, read the appropriate sections before making your selection.

☒ Life sciences ☐ Behavioural & social sciences ☐ Ecological, evolutionary & environmental sciences

For a reference copy of the document with all sections, see nature.com/documents/nr-reporting-summary-flat.pdf

Life sciences study design

All studies must disclose on these points even when the disclosure is negative.

Sample size	Sample size was chosen based on our prior studies using the same types of assays, as well as our previous publication, to ensure statistically significant results. All experiments was preformed at least three times or with at least three biological replicates.
Data exclusions	For the experiments in our study, no data was excluded.
Replication	Quantitative real-time PCR, western blot, luciferase activity assay, chromatin immunoprecipitation (ChIP), co-immunoprecipitation, colony formation assays, EdU assay, Transwell assay, wound-healing assay and Hoechst 33258 staining assay were performed with at least three independent experiments. All attempts at replication were successful. Animal studies, in situ hybridization, immunohistochemistry were performed with biological replicates.
Randomization	Mice were randomized to treatment groups. Samples of patients with colorectal cancer or gastric cancer were randomly collected for quantitative real-time PCR, western blot, in situ hybridization and immunohistochemistry experiments
Blinding	The experiments were not blinded as the experimental observations would be irrespective of blinding. Conclusions were made based on independent experiments, biological replicates and significance of the data.

Reporting for specific materials, systems and methods

We require information from authors about some types of materials, experimental systems and methods used in many studies. Here, indicate whether each material, system or method listed is relevant to your study. If you are not sure if a list item applies to your research, read the appropriate section before selecting a response.

Materials & experimental systems

n/a	Involved in the study
☐	☒ Antibodies
☐	☒ Eukaryotic cell lines
☒	☐ Palaeontology and archaeology
☐	☒ Animals and other organisms
☒	☐ Clinical data
☒	☐ Dual use research of concern
☐	☒ Plants

Methods

n/a	Involved in the study
☒	☐ ChIP-seq
☒	☐ Flow cytometry
☒	☐ MRI-based neuroimaging

Antibodies

Antibodies used	All the antibodies used in this study have been described in detail in Materials and Methods.
Validation	All commercially available antibodies were validated by the suppliers. Details can be referred on the manufacturers' websites.

Eukaryotic cell lines

Policy information about cell lines and Sex and Gender in Research

Cell line source(s)	The cell lines sources have been described in the section of Materials and Methods.
Authentication	All cell lines were authenticated using STR analysis.
Mycoplasma contamination	All cell lines tested negative for mycoplasma contamination.
Commonly misidentified lines (See ICLAC register)	No commonly misidentified cell lines were used.

Animals and other research organisms

Policy information about studies involving animals; ARRIVE guidelines recommended for reporting animal research, and Sex and Gender in Research

Laboratory animals	Four- to six-week-old BALB/c-nu/nu nude female mice were used for animal studies.
Wild animals	No wild animals were used in this study.
Reporting on sex	Female mice were used in our study.
Field-collected samples	No field-collected samples were used in this study.
Ethics oversight	All studies involving animals were conducted with the approval of Southern Medical University Experimental Animal Ethics Committee.

Note that full information on the approval of the study protocol must also be provided in the manuscript.
